# The Impact of Cost-Containment Schemes on Outpatient Services for Schoolchildren with Refractive Errors in Taiwan—A Population-Based Study

**DOI:** 10.3390/children9060880

**Published:** 2022-06-13

**Authors:** Koyin Chang, Wen-Li Lee, Yung-Hsiang Ying

**Affiliations:** 1 Department of Healthcare Information and Management, Ming Chuan University, Taoyuan 33348, Taiwan; kychang@mail.mcu.edu.tw (K.C.); wllee@mail.mcu.edu.tw (W.-L.L.); 2 Department of Business Administration, National Taiwan Normal University, Taipei 10617, Taiwan

**Keywords:** myopia, global budgeting, health inequity, demand elasticity

## Abstract

Objectives: Extant research on cost-sharing finds no impact on health care utilization when the amount is insubstantial. This research investigates the effects on nonacute outpatient services for schoolchildren with refractive errors in Taiwan and discusses the potential harm caused by cost sharing and relevant cost containment policies. Methods: Longitudinal claims data from the National Health Insurance database are employed. District demographic information is also used for aggregate-level analyses. Interventional modeling is conducted on pooled individual-level data with a Poisson model and negative binomial models. Generalized least square modeling is performed on aggregate district-level data to elucidate the impacts of cost sharing and the reimbursement rate with controls for patient and institutional characteristics, district socioeconomic factors, and competitiveness among institutions. Results: The findings of this study show that cost sharing does not significantly affect children’s utilization of outpatient services in the patient-level analyses. However, it significantly decreases the service volume based on the results of district aggregate analyses. There are potentially marginal patients in society, and they are more likely to be girls in poorer families, whose chances of seeking medical care significantly decrease when cost sharing increases. Conclusions: The gap in health inequity can be widened when stringent cost-containment policies are implemented. The offset effect caused by delayed care may also result in higher health care expenditures later. Cost sharing for children should be separately and prudently designed to better protect them from deprivations caused by changes in health policies.

## 1. Introduction

The notion that an increase in cost sharing can reduce the usage of health care services has been well established since RAND cooperation conducted its health insurance experiment (HIE) in the 1970s (Manning et al. 1987; Newhouse, 1993) [1,2]. Global budgeting (GB) for the health care system has also proven to be effective in lowering inflation-adjusted health care spending by 9–17% [3,4,5,6]. Following the cost containment schemes adopted by many Western countries, the Taiwan National Health Insurance Administration (NHIA) has employed various methods to reduce the ever-increasing cost of national health expenditures. Under the global budget (GB), these methods include the adjustment of copayments and diagnosis-related groups (DRGs). However, the increase in health care expenditures continues unabated. There may be a cross-price substitution effect: A decrease in outpatient visits resulting from rising cost sharing could cause an increase in hospital utilization or more expensive drug spending, resulting in an offset effect on total health care expenditures [7]. Delays in obtaining treatment due to higher costs could also exacerbate patients’ disease conditions and lead to higher medical costs later on.

However, studies on cost sharing based on Taiwanese data suggest that user fees are unlikely to change patients’ care-seeking behavior, meaning that patients are insensitive to price changes [8,9]. Those studies mostly focus on acute diseases such as cardiovascular diseases [10] and pneumonia [11] or on smaller-scale effects in contexts such as individual hospital settings [12,13]. The dynamics of nonacute care, a relatively elastic service on both the demand and supply sides, have not yet been elucidated. Since treatments for different diseases have different values, common forms of cost sharing built into insurance plans may not commensurately align one with another [14]. Thus, this research aims to investigate the utilization of health care services among schoolchildren with refractive problems, a condition that is nonacute in nature, with a focus on vulnerable groups. The quasi-experimental design enables more transparent inspection of changes in patients’ care-seeking behavior and physician practice behavior under policy changes.

Refractive errors are common vision problems around the world. Myopia, in particular, may be a simple, minor condition for many people. However, for many others with serious eye disorders such as cataracts, glaucoma, or maculopathy, myopia is the root of diseases that can lead to blindness [15,16,17]. Indeed, high myopia maculopathy is the leading cause of irreversible blindness in many countries around the world [18,19,20,21,22,23]. Taiwan’s myopia prevalence is especially high at over 80% [24], as opposed to approximately 40% in the United States [25] and to the world average of 50% [26]. This condition is prominent for schoolchildren in particular and has been on the rise. [27]. Previous research suggests that if a child’s myopia can be kept from progressing, the risks of contracting myopic maculopathy, retinal detachment, and posterior subcapsular cataracts would be 4 times, 3 times, and 1.5 times lower, respectively, than the risks for a child with myopia progression [28]. Strong evidence suggests that something more fundamental than genetics or local environmental factors is at work in influencing myopic progression [29].

### 1.1. Schoolchildren Vision Care Program

The high prevalence of myopia among schoolchildren in Taiwan has drawn public health authorities’ attention. Continuous efforts to prevent myopia have always been an important public policy for both the Ministry of Education (MOE) and the Ministry of Health and Welfare (MOHW) of Taiwan. Approximately 30 years ago, the MOE initiated the Taiwan Student Vision Care Program (TSVCP) to fight myopia. Nationwide student vision screenings were performed by school nurses on a regular semester basis. If students failed to meet the 20/25 threshold (Snellen visual acuity of 20/25 or less), they receive a follow-up eye examination slip that has to be signed by an ophthalmologist and returned to school nurses [29] (Other measures funded by government agencies to enhance students’ vision are referred to in Wu et al. (2018).). Shih et al. reported the effectiveness of atropine on myopia progression in 1999 in a study in which referred children were prescribed atropine eye drops; the drops slightly lowered the prevalence of high myopia for 18-year-olds from 20.8% in 2000 to 16.9% in 2006 [30]. Since then, parents in Taiwan have had an incentive to take their myopic children to ophthalmologist offices in the clinics or hospitals for repeated follow-up visits after the initial screening. To access vision care from ophthalmologists, prior appointments are required. Alternatively, sometimes parents may take children to optometrists for follow-up examinations in optical shops, where walk-in services are possible and no fees are required since their revenue is from selling eyeglasses, not providing eye care services. The nonacute nature of refractive errors provides a scenario in which the patient demand for medical care services is relatively elastic. The phenomenon of “marginal patients”—patients, usually members of disadvantaged groups of people who tend not to receive health care unless the situation is critically serious—could be widespread. Thus, the impact of policy changes in Taiwan’s insurance system on nonacute outpatient services can be elucidated.

### 1.2. NHI Cost Containment Scheme

#### 1.2.1. Global Budgeting

The premise of GB systems, namely, that health insurance reimbursement is capped at a predetermined annual amount, has proven to be an effective way to contain ever-increasing health care expenditures [4,11,12,13]. Providers have an incentive to cut unnecessary health care services but sometimes may put health care quality at risk [10,12]. In the wake of the large cost increases after the implementation of the National Health Insurance system, Taiwan phased in GB in 1998 starting with dental care. Then, in July 2001 and July 2002, GB was expanded to clinical (ambulatory care) and hospital services, respectively, which are two sources of primary care provision for schoolchildren’s eye care and treatment.

Five global budget sectors were designated: hospital care, private clinical care (primary ambulatory care), dental care, traditional Chinese medicine, and renal dialysis. Under each of the common global budgets, physicians maximize their share by expanding their volume of services, in a zero-sum game [31]. The NHIA sets national uniform reimbursement rates (points) for each of the five sectors each quarter. A basic relative-value schedule assigns different points to each health care service; these points are then translated to a monetary fee schedule at the end of each quarter. Under the global budgets, the point values “float”, or automatically adjust, based on the total volume of services. One point is targeted to be equal to 1 new Taiwan dollar (TWD) and usually fluctuates in the range of approximately TWD 0.80–0.95 (USD 1 = TWD 30.5 in 2015). The adjustment is usually downward so that total payments to providers within the different sectors in each quarter do not exceed their common budget. In refractive error treatment, the reimbursement for outpatient services is 90–352 points, depending primarily on what class the institution is in (for example, hospital or clinic), on whether prescriptions are released to outside pharmacies, and on how many patients are seen on a given day. The NHIA penalizes physicians for providing excessive outpatient services by reducing service reimbursement. The reimbursement is on a downward sliding scale with brackets of 30, 50, 70, and 150 patient visits per day (MOHW 2018). The fee schedules also apply differently across geographic regions, considering the differences in population age structure, mortality rate, utilization rate, and general price level across regions.

#### 1.2.2. Changes in Cost Sharing

Copayment, which is set by the NHIA, is charged alongside registration fees by health care providers when patients receive outpatient consultation services. Registration fees vary across individual providers and usually range from TWD 20 to TWD 300, depending on the pricing policies of each facility, which is related to the geographic location and level of facilities. Meanwhile, copayment is tiered and regulated by NHIA to steer patients toward seeking services from local clinics and district hospitals in lieu of medical centers or regional hospitals where tertiary and secondary care are provided. There have been several copayment policy reforms in the past 20 years, including copayment changes for inpatient services, ambulatory care, referral outpatient services, nonreferral outpatient services, prescription medication services, lab test services as well as high-user surcharges for outpatient services (Ministry of National Health Insurance, 2011). A detailed description of the copayment adjustments is provided in Appendix A (Table A1).

This study adopts a broad definition of cost sharing, including copayment, registration fee, and insurance premium. The extant research rarely discusses the impact of premium adjustment because it is considered a sunk cost and not relevant to the decision making of patients. However, in Taiwan, the premium is a type of payroll tax, and the premium rate (fee rate) is floating to let NHIA maintain a balanced budget. Higher NHI expenditures imply higher rates in the future. As a result, an upward adjustment in the premium rate may signal to the insured that they to conserve the utilization of health care.

### 1.3. Incentives for Providers

Under the GB umbrella of Taiwan’s National Health Insurance (NHI) system, incentives for physicians are dynamically subject to the total volume of services provided in different regions and different classes of health care institutions. The revenue sources for Taiwan’s providers are threefold: NHI reimbursement, copays and registration fees from patients, and self-pay services and products [32].

With the budget capped and fee-for-services as the predominant payment scheme, there is fierce competition among health care providers for patients, and thus, return visits are important. Except for the copayment and registration fee for each visit, there is no out-of-pocket cost for insured patients who receive services in the care categories approved by the administration of NHI. An ophthalmologist’s monthly revenue, therefore, is primarily dependent on the total number of service points in a month and the dollar value of each point in the region in which the provider is located. The dollar value of each service point is recalculated each quarter based on the region’s total points claimed during that period. Restricted by the predetermined expenditure cap, a region that has a higher service volume has a lower dollar value per service point and vice versa. Thus, reimbursement rates for consultation services are floating and differ across regions depending on the allocated budget, the number of services provided, and the practice patterns of physicians in a region. Physicians’ practice is guided accordingly when incentives change. The research focus here is threefold: the extent to which service volumes for nonacute conditions are affected by the point values of GB reimbursement, the changes in cost sharing, and the competitiveness among providers within a locality.

## 2. Materials and Methods

### 2.1. Data Sources

The data in this study are from the NHI database as documented and released by the MOHW, which provides claims data and provider and patient background information. Since the NHI covers over 99.5% of legal residents in Taiwan, it is the most representative health care database available for the country. Two million randomly sampled beneficiaries were drawn from the entire population of Taiwan in 2010. Their claim information is traced back to 2000 and forward to 2017 to form the longitudinal structure. In this study, we only use data up to 2015 before the International Classification of Diseases code switched from the ninth version to the tenth. The primary diagnoses for each outpatient visit in the NHI database are assigned a code based on the ICD-9 code. This study focuses on schoolchildren with refractive problems, predominantly myopia (ICD code 367.1). Also included are astigmatism (367.2) and amblyopia (368.0). The various forms of utilizing outpatient services are the outcome measure; however, the services are not exclusive to ophthalmologist office visits; thus, the observed service volumes increase to include more comprehensive results for the patients’ general health conditions). Since the point values float every quarter, the observation frequency is quarterly in the study. In addition to individual patients as the observed units, data are also stratified by the health care provider and geographic location for analysis.

### 2.2. Charlson Comorbidity Index (CCI)

Patients who are less healthy tend to pay more than healthier people for physician visits. Thus, each patient’s health status has an impact on the utilization of health care services. To establish an objective measure of each patient’s health condition, the Charlson comorbidity index (CCI) is adopted to capture patient comorbidity. The CCI is a method of categorizing patient comorbidities based on ICD diagnosis codes. Each comorbidity category has an associated weight (from 1 to 6), and the sum of all the weights results in a single comorbidity score for a patient. A score of zero indicates no comorbidities. The higher the score is, the more likely the predicted outcomes of mortality or higher resource use. Over time, there have been changes to the index presented in different studies. The original index was developed with 19 categories [33], but it has been modified to include 17 categories [34]. The impact of the CCI can be twofold. A positive index suggests that unhealthy patients utilize health care services more often, while a negative index may reflect patient substitution; that is, other disease conditions may decrease patient demand for vision care [35,36,37].

### 2.3. Herfindahl-Hirschman Index (HHI)

Health care utilization can be affected by providers’ attitudes. In particular, competitiveness among providers may be related to the strategies of institutions; for example, physicians may seek to manipulate the frequency of patient follow-up visits, resulting in a so-called supplier-induced demand (SID). The Herfindahl-Hirschman index (HHI) is a commonly accepted measure of market concentration. It is calculated by squaring the market share of each firm competing in a market and then summing the resulting numbers. It can range from close to zero to 10,000. The closer a market is to being a monopoly, the higher the market concentration (and the lower the level of competition). In the market for myopia care, market share is calculated by outpatient/physician office visits within the year in each zip code defined by the NHIA. A similar method has been employed previously in contexts where there is no published HHI available for specific health care services [38].

### 2.4. Empirical Strategies

Our empirical analyses are twofold. First, individual-level analyses are conducted with patients as the observation unit and facilities as the observation unit, and second, district (cities/counties) aggregate-level analyses are conducted for patient utilization and facility services provided.

#### 2.4.1. Individual-Level Regression Analyses–Pooled Data

Quarterly count data for physician office visits are obtained for children with refractive errors in our sample. The data are counted at two different levels, patient and institution (office visits by physicians are not analyzed due to the lack of complete physician information in the database). At the patient level, the panel regression method is not suitable since each patient does not always have annual office visits throughout the entire 16-year study period. As a result, each period is composed of a large number of very different patient groups. For this reason, we use the pooled data method instead of the panel regression method.

In addition to the ordinary least squares (OLS) method, the Poisson model and negative binomial (NB) regression are also employed. OLS assumes that the variation in the dependent variable at each level of the regressor is the same; however, this is usually not the case for count data. Poisson regression, on the other hand, allows the responses at each level of the regressor to become more variable with increasing means. The Poisson model is subject to the highly restrictive assumption that the variance is equal to the mean given by the model. Based on the Poisson-gamma mixture distribution, NB is popular because it models Poisson heterogeneity with a gamma distribution [39].

##### Patient Unit of Analysis

Following the analysis model of Chandra et at. (2010) [7], the quasi-experimental change in the NHI GB (global budgeting) policy takes the form of:

(1) $$UTIL_{pt}=\beta_{0}+\beta_{1}Point_{pit}+\beta_{2}Copay_{t}+\beta_{3}HHI_{it}+\beta_{4}Time+\beta_{5}X_{pt}+\delta_{i}+\varepsilon_{pt}$$

The patient is the unit of analysis. *UTIL* is a measure of utilization (or total outpatient visit count) per patient *p* at time *t* in district *i*, Point is the reimbursement value after the implementation of GB, and $\delta_{i}$ is the district fixed effect. HHI is the Herfindahl-Hirschman index of the health care provider used by patient *p* in district *i*. The *Xs* are patient background variables such as gender, age, parent income level, parent job classification, and CCI, that is, patients’ comorbidity condition in that quarter. Time captures changes in general social norms and health and economic conditions. The regression coefficient is denoted by *β*, and *ε_pt_* is i.i.d. with normal properties, i.e., The effect of the policy change is identified by *β*_1_, which measures the change in the utilization relative to the change in the reimbursement value within provider classifications, and *β*_2_, which measures the change in cost sharing of the patients. In Taiwan, patients can freely choose their physicians and health care providers. Although patients may seek services across regions, their behaviors are assumed to be most relevant to their residential location. Thus, the Point and HHI used in the analyses are based on the patients’ location, not the providers from whom they obtain services. Copay is charged differently for different categories of providers, and this amount is averaged out from the quarterly visits of the patients.

##### Institution Unit of Analysis

In the second model, the institution (provider) is the unit of analysis. The counts of refractive error outpatient services provided by each institution serve as the outcome variable (Service):

(2) $$Service_{fit}=\beta_{0}+\beta_{1}Point_{it}+\beta_{2}Copay_{ft}+\beta_{3}HHI_{it}+\beta_{4}Time+\beta_{5}X_{ft}+\delta_{i}+\varepsilon_{ft}$$

The institution characteristics (*X_ft_*) of facility *f* in district *i* at time *t* include the institution level (medical center, hospital, clinic, etc.), institution age, gender of the facility CEO, and institution scale, as measured by the facility’s total outpatient services, total number of physicians, total number of full-time ophthalmologists, and total number of part-time ophthalmologists. (The numbers of full-time and part-time ophthalmologists are readily available in the databank. Full-time members are directly hired employees, while part-time members usually work in the facility for less than 20 h a week.)

#### 2.4.2. Generalized Least Squares Method-Aggregate District Data

The aggregate amounts of outpatient services received by patients and provided by institutions are further calculated on the basis of 20 districts (cities/counties) in Taiwan, as presented in Equations (3) and (4), respectively. The results are then submitted for GLS analyses to clarify the extent to which variations in the amounts of outpatient services are determined by health policies such as the copayment and insurance *fee* changes, the district income level, the amount of health resources, and demographic characteristics:

(3) $$UTIL_{pit}=\beta_{0}+\beta_{1}point_{it}+\beta_{2}Copay_{t}+\beta_{3}Fee+\beta_{4}NHCC_{it}+\beta_{5}Time_{}+\beta_{6}X_{it}+\delta_{i}+\varepsilon_{pt}$$

where *UTIL* is a measure of utilization (or total counts of outpatient visits) for patients living in district *i* at time *t*, point is the *point* value for reimbursement with the implementation of GB, and $\delta_{i}$ is the district fixed effects. *NHCC*, which is the number of health care facilities in the district, reflects competitiveness among providers in district *i*, and *Xs* are the district socioeconomic and demographic background variables such as the average household income level in the district, population density, number of health care providers, and educational background of residents. *Time* represents the time trend, which captures changes in general social norms and health and economic conditions. The regression coefficients are denoted by *β*, and *ε_pt_* is i.i.d. with normal properties, i.e., outpatient services can be aggregated based on the volume provided by institutions in each district. The estimated measure becomes:

(4) $$Serv_{fit}=\beta_{0}+\beta_{1}Point_{it}+\beta_{2}Copay_{ft}+\beta_{3}Fee_{it}+\beta_{4}NHCC_{it}+\beta_{5}Time+\beta_{6}X_{t}+\delta_{p}+\lambda_{t}+\varepsilon_{pt}$$

where *Serv_fit_* is the total number of services (or total outpatient visit count) provided by institutions located in district *i* at time *t*. The control variable *Xs* are the same socioeconomic and demographic variables as described in Equation (3).

## 3. Results

From the NHI outpatient service claim dataset, we retrieve the information for children under 18 years of age from 2000 through 2015. The summary statistics for these children and for the vision care facilities over the whole sample period and in selected years are presented in Table 1 and Table 2, respectively. The left-hand side panel of Table 1 presents data for all children who had at least one physician visit in the sample, and the right-side panel contains data for children who have sought care for refractive errors. The number of physician visits is measured quarterly, and income is the monthly proxy figure derived from the insurance premium rate. There are more female than male schoolchildren in the myopic group, especially in earlier years, but their percentage decreases to approximate parity. The average age of the children with refractive errors is approximately the same throughout the study period, but it very gradually increases because some patients are repeatedly sampled over time, and no patients in the sample were born after 2010 due to the data limitation. Children in the two groups show similar numbers of quarterly outpatient visits and CCIs, suggesting that their general health conditions are about the same. Children with refractive errors make an average of 4.23~3.42 physician office visits in a quarter, not exclusive to ophthalmologist visits.

**Figure 1:**
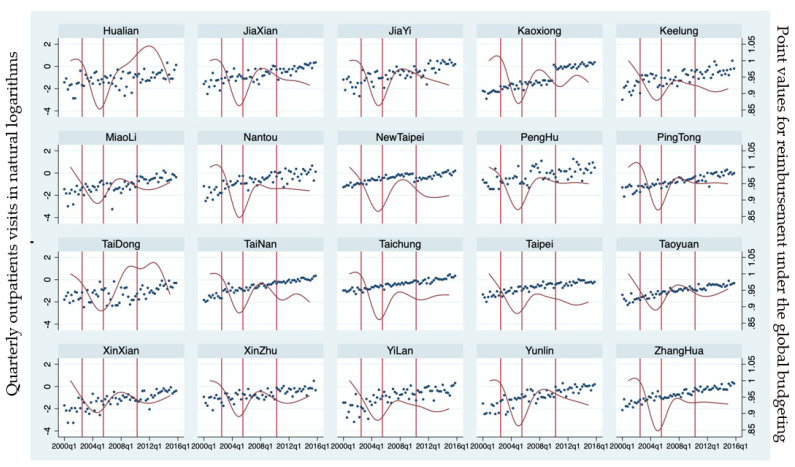
Total Outpatient Visits of Children with Refractive Errors by Patient Residential District. Note: Dots represent the quarterly volume of total outpatient visits per thousand children with refractive errors in each district in natural logarithms (the scale on the left-side vertical axis), and red lines denote the point values for reimbursement under the global budgeting system with the scale on the right-side vertical axis. The vertical lines represent insurance fee changes.

**Figure 2:**
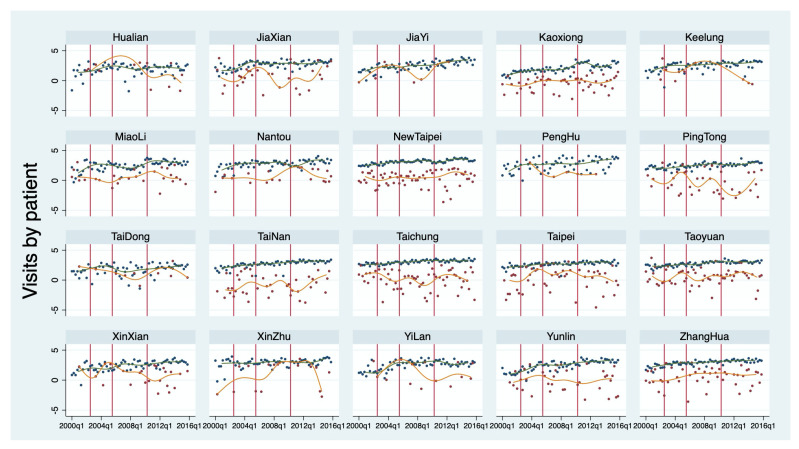
Total Outpatient Visits of Children with Refractive Errors by Institution Type and District. Note: Dots represent the quarterly number of total outpatient visits of children with refractive errors provided by type of facility in natural logarithms. Blue and orange denote clinics and hospitals, respectively. The smooth lines are the corresponding cubic splines. The vertical lines represent fee changes.

To obtain a general picture of the utilization of children’s outpatient services, the outcome variables are drawn against time and policy changes, as shown in Figure 1 and Figure 2. The point value appears on the scale on the right-side axis in Figure 1. The changes in copayment schedules (the first two vertical lines) and premium rates (the third vertical line) seem to not have an impact on utilization by children with refractive errors. Figure 2 breaks down the services by type of institution (clinics and hospitals) and shows that more outpatient services are provided in clinics than in hospitals. Both figures suggest a moderate increasing trend in all districts in the number of physician office visits over the study period, which suggests that the time trend (Time) variable is important to include in the regression analyses to capture the phenomenon.

### 3.1. Individual-Level Analyses–Outpatient Services

Regression analyses for patient-level data, as in Equation (1), are presented in Table 3, in which the ages of both patients and institutions take the form of polynomials of degree two. All three models (OLS, Poisson, and NB) exhibit comparable results: Boys tend to utilize more services than girls, and income and comorbidity are also positively associated with a higher level of utilization. Age, the competitiveness of the institution’s market (or HHI), and insurance premiums are negatively associated with health care utilization. All results are statistically significant at the 1%, 5%, or 10% level. Seasonal effects are also proven to be impactful, with quarters 1 and 4 (the start of the semester, when school screenings are at their peak) showing more service utilization than other quarters. Children with a vulnerable health status exhibit especially high utilization. Those categorized as suffering from a natural injury and those needing respirators utilize outpatient services more than those in other categories. Lastly, the point value and copay do not show significant impacts on outpatient service utilization, implying that the level of utilization is not dampened by rises in copays or adjustments of the point value.

Table 4 presents outpatient services provided by institutions, according to the panel model estimation of Equation (2). The age of the institution, competitiveness of the market, and total number of physicians in the institute exhibit negative impacts on the number of outpatient services provided. Medical centers provide more services than nonmedical centers, and clinics provide more services than hospitals in general. The point values and changes in copayments have a significant impact on the service level only in the Poisson model. Other drivers of the service volume are primarily the scale of the institution: The greater the number of total outpatient services provided and the greater the number of full-time ophthalmologists in the institution, the more refractive error services are supplied.

### 3.2. Aggregate Data Analyses–Count Data of Outpatient Services

To understand the geographic variation in health service utilization, the aforementioned outcomes measured are aggregated to the district level and then analyzed in relation to district socioeconomic and demographic characteristics, with controls for the population density and the density of health care institutions in each district. The results are presented in Table 5. The point value, insurance premium rate (fee), and copay are all negatively associated with service utilization by patients (Column 1). Copay and premium rate changes do not significantly impact the change in the volume of total services provided by institutions in a district, which are driven by the hospital density (NHCC), as shown in Columns 2~4. Lastly, the impact of income on the outpatient service utilization exhibits a negative relationship in all measures, in contrast with what is found in the individual patient-level analysis.

### 3.3. Robustness Tests

To ensure the robustness of the estimation, all regressions have been tested by various functional forms by adding and dropping different variables in the regressions. Polynomial forms or log linear forms of the functions are tested based on past econometric experiences. The income, population density, population ratio with a college degree, and institution density are highly correlated, which might cause some concerns about collinearity problems. However, the results do not change significantly when excluding one or some of the variables from the regressions. The details and process are not reported to conserve space.

To ascertain the robustness of our results, we also collect the expenditure data on the outpatient services from the patients’ perspective and the corresponding revenue from the institutions’ perspective. The analyses are reported in Table 6. All the signs of the covariates are the same as those in Table 5, except for Copay-1 and Copay-2. The positive effect of copays is as expected since increases in the amounts of copayments represent direct cost sharing by patients and are injected into the institutions as revenue. The comparable results of Table 5 and Table 6, along with the aforementioned empirical practice, suggest that the estimation models are reasonable and generate robust results.

## 4. Discussion

Since the Taiwanese government started annual vision checkups for all children on school campuses in 1999, parents have grown accustomed to seeking follow-up eye examinations from ophthalmologists in health care institutions if their children do not meet the 20/25 threshold. With the effectiveness of atropine reported by Shih (1999), parents have an incentive to routinely take their children back to ophthalmologist offices to receive the prescription drug. The treatment of myopia addresses a chronic condition, and the aim is to slow down the progression. This nonurgent health care service provides a scenario for understanding the medical-care-seeking behavior of patients and practice pattern of physicians under different fee policies.

Based on NHI claim data from 2000 to 2015 for two million randomly selected residents of Taiwan, our results show that the participating health care facilities become younger and provide smaller volumes of services on average over time (as shown in Table 2). The competitiveness has decreased in the past two decades (larger HHI), which suggests that facilities may be gaining larger market power. A greater share of facilities were clinics, instead of hospitals, in 2015 than in 2000. For patients’ care-seeking behavior, our data suggest that children of younger ages tend to seek more outpatient services than children of older ages, and boys utilize more services than girls, with statistical significance at the 1% level in all models employed in the research, implying that gender bias persists in Taiwanese society. Residents living in districts with a lower population density seek fewer services, and a plausible explanation is that a more spacious living environment is related to better vision among children, which is consistent with the extant research [31]. The comorbidity index is positively related to children’s utilization of outpatient services, which suggests that children with weaker health conditions are generally seeking more health care services.

In terms of cost sharing, changes in copayments do not significantly affect the health care utilization. However, the insurance premium is negatively associated with service utilization at the 1% significance level in the patient-level data. When we further analyze district-level data, the total number of physician office visits in the aggregate patient data also decreases when higher premium rates are charged and higher surcharges for nonreferral visits are implemented. On average, our findings suggest that a 1% increase in the premium rate decreases the outpatient visits of children by 8.84%, and the implementation of higher cost sharing in nonreferral visits negatively affects the care-seeking behavior of patients by 20.22% (the coefficient 0.112 divided by the mean values of district number of visits per thousand children, 0.5542). The result shows a greater effect than that found in the existing literature [40], where single-year data were analyzed for all outpatient visits using Taiwan NHI data. For institutional outpatient service analyses, the impact of copays on the volume of services is mixed; however, the aggregate institutional revenue is positively affected, which is as predicted since higher copays from patients go directly to institutions’ revenue. We further decompose the aggregate institutional data into clinic and hospital aggregate data. Our findings show that the impact on revenue is significantly positive for clinics but insignificant for hospitals, suggesting that higher copays lead patients to seek more services from clinics and fewer from hospitals (Columns 3 and 4 in Table 6). This result suggests that policies can provide effective incentive to direct patients’ care-seeking behaviors, achieving the goal that patients should seek care for nonurgent noncritical health conditions at primary care facilities (or clinics) and leave the care of more acute and critical conditions to hospitals.

Comparing the results between the services of clinics and hospitals, the former are more susceptible to district characteristics and policies changes. Hospitals are insensitive to changes in copayments, premium rates, and point values under the GB system. On the other hand, clinics’ volumes of services provided are negatively related to the point value, and their revenues are positively associated with copayments at the district aggregate level, indicating that clinics are more responsive to policy changes. The negative HHI coefficients suggest that health institutions with greater market power tend to provide fewer outpatient services. Competitiveness among health care institutions is directly linked with a greater volume of services, implying the existence of SID for medical services.

Finally, income has a positive effect on the outpatient service utilization in patient-level analyses, implying that patients from disadvantaged families tend to under-utilize health care services. The phenomenon of marginal patients could be prevailing. Also suggested is that the parents of the disadvantaged families might seek follow-up services for their children from optometrists in optical shops where walk-in service is possible without a medical fee. Even though the signatures of optometrists on follow-up slips are normally not accepted by TSVCP, the regulations are not strictly enforced because doing so would impose extra burdens on school nurses. In district-level analyses, the effect of income turns negative. The explanations for this phenomenon can be twofold. First, it could be that people who live in wealthier districts tend to be healthier and demand fewer health care services. Another possible explanation is that people in wealthier district utilize somewhat different vision care services than traditional mydriatic eyedrop treatments. For example, vision correction surgery (LASIK) and orthokeratology contact lenses are gaining increasing popularity in Taiwan for containing myopia progression. Both are not covered by Taiwan NHI and, hence, are not recorded in the NHI database. Even though patients who have undergone refractive surgery or orthokeratology still need to be followed up closely for the risk of posterior segment alterations, those patients often receive eye care from the same ophthalmologists who opt out of NHI. Hence, they would show few or no follow-up outpatient visits.

## 5. Limitations

There are limitations to this study. First, the percentage of schoolchildren who seek vision care services through NHI is only approximately 30% in our sample, which is far lower than that reported by the Taiwan Health Promotion Administration [7], according to which the myopic rates are 48.8% and 71.6% for children in the age groups of 7~11 and 12~18, respectively. This phenomenon suggests that only a proportion of myopic children seek vision care through the Taiwan NHI. The children who do not seek vision care through the NHI could be either from wealthy families who receive alternative treatments or from disadvantaged families who do not seek treatment at all, the so-called marginal patients. They might obtain services from optometrists for follow-up examination, which is a cheap substitute for ophthalmologists’ vision care. As a result, the NHI records show a much lower follow-up rate than what was reported by government authorities. Our research cannot distinguish these two groups. Second, our data range from 2000 through 2015, and the sampled patients are based on the population in 2010, i.e., no patients were born after 2010 in our study. We do not foresee too much impact from this problem, since myopia usually occurs in children after they enter grade school. Third, due to the limitation of data, we also lack information before 2000, i.e., the pre-intervention period of GB implementation. The best method of circumventing the data limitation is to study the impacts of changes in copayment and premium when investigating the impact of cost sharing, as adopted in other studies [7]. Additionally, this study does not distinguish between physician visits in general and those related to the child’s vision issues. Future studies with this specific analysis objective might be of interest for exploration. Finally, the NHI database does not provide service quality indicators and is held by the Taiwan MOHW, which only allows research and analyses to be performed on site at the designated location using its computing equipment. More advanced econometric analyses cannot be performed due to the restrictions on data and equipment availability and access.

## 6. Conclusions

The notion that an increase in cost sharing reduces the usage of health care services has been well established since RAND conducted its HIE in the 1970s [1,2]. Past studies on cost sharing based on Taiwanese data, however, suggest that user fees are unlikely to change patients’ care-seeking behavior, meaning that patients are insensitive to price changes [8,9]. The consensus is that the amount of fee change is not sufficiently substantial to cause impact. This study focuses on patients with refractive errors and nonacute diseases in general, not necessarily the treatment of their refractive errors. The advantage of focusing on children is that parents rather than the patients themselves are the payers of the services, and myopia in children does not cause immediate serious harm, providing a low or delayed incentive for parents to take their children to ophthalmologists’ offices for routine follow-up treatment. Our outcome measures are various forms of total outpatient services utilized by myopic children but not exclusive to ophthalmologist services. Thus, a greater pool of observations was obtained for more general understanding. Consistent with the previous literature, the results of our study indicate that the change in copayment does not significantly affect the outpatient visit behaviors in individual patient data, which suggests that children who continuously seek health care services do not alter their care-seeking behavior when the amount of copayment simply varies. However, our data indicates that only approximately 30% of children seek vision care services, which is far below the myopia prevalence rate in children reported by the Taiwan Health Authority. This phenomenon suggests that there are “marginal patients” who do not receive services or quit receiving services when the cost is considerably high. This phenomenon is prominent when analyzing aggregate district data; significantly fewer outpatient services are provided after cost sharing increases in the form of copayment or premium increases. In particular, the implementation of higher cost sharing in nonreferral visits decreases the care-seeking behavior of schoolchildren by as much as 20.22%.

For the effect on the practicing behavior of providers, competition is the key factor in determining their volumes of services. The HHI has a significantly negative impact, i.e., greater competition induces greater volume of services provided. However, when decomposing the effect to the hospital and clinic levels, only the latter shows a conspicuous effect, indicating that clinics are more susceptible to policy changes and possibly more incentive driven than hospitals.

In conclusion, this study provides implications for the relevant authorities regarding the effect of cost sharing on patients’ utilization of care for nonacute chronic conditions. While the fee adjustments may not be substantial and individual patients may not change their care-seeking behavior, the effects at the aggregate level can be significant, considering that more patients do not seek services at all. The cost savings in the short run might thus generate higher medical spending in the long run. In addition, treatments for different diseases have different values, and common forms of cost sharing built into the insurance plans may not align socially optimal outcomes with privately optimal ones [14]. Thus, cost-sharing schemes should prudently differentiate based on the treatment values and patients’ backgrounds. For example, cost sharing for children can be separately designed to better protect them from disadvantages caused by the changes in health policies.

## Figures and Tables

**Table 1:** Summary Statistics for All Children and Children with Refractive Errors.

	All Children			Children Refractive Errors		
	2000	2008	2015	2000	2008	2015
Phy. visits	4.28 (2.31)	3.80 (2.02)	3.21 (1.07)	4.23 (3.07)	3.76 (2.89)	3.42 (2.70)
Income (,000)	21.99 (12.40)	27.43 (19.50)	30.41 (22.67)	23.90 (12.90)	29.47 (20.73)	31.86 (23.21)
Premium	1253.14 (751.61)	1560.92 (1051.72)	1711.22 (1304.46)	1336.34 (825.75)	1650.57 (1112.99)	1781.10 (1273.79)
Age	9.74 (5.53))	11.08 (5.12)	12.27 (4.02)	10.74 (3.8)	10.38 (3.60)	11.27 (3.48)
CCI	0.26 (0.43)	0.27 (0.43)	0.29 (0.45)	0.25 (0.53)	0.25 (0.51)	0.27 (0.56)
Boy	49.2%	50.6%	51.40%	45.77%	49.61%	50.01%
Myopia%	14.58%	23.71%	29.38%			
Parent Employment			Pub. Emp	12.70%	10%	7%
		Pub. sector		2.13%	1%	1%
		Private sector		41.67%	45%	47%
			Farmer	13.89%	13%	10%
			School	2.41%	3%	3%
		Self-employed		7.41%	9%	9%
Obs. No	433,912	386,208	351,792	10,238	21,844	18,812

Note: Standard deviations are in parentheses. Data are counted from two million sampled patients. Only schoolchildren younger than 18 years of age are included. All dollar measures are in new Taiwan dollars (TWD). Physician visits and health expenditures are quarterly measures. Income is the proxy of monthly income based on the insurance premium level released by the NHIA. The CCI is the Charlson comorbidity index.

**Table 2:** Summary Statistics for Vision Care Facilities.

		All Types			All Years	
	2000	2008	2015	All	Clinics	Hospitals
Instit. Age	20.99	15.63	13.04	15.70	15.70	15.78
	(6.73)	(7.91)	(8.17)	(8.04)	(8.03)	(8.39)
Volumes	190.15	124.48	81.58	126.98	116.90	422.00
	(563.85)	(183.69)	(140.44)	(236.80)	(163.00)	(909.30)
HHI	1.89	4.49	3.60	2.90	2.95	1.34
	(24.01)	(102.52)	(33.48)	(63.12)	(64.18)	(4.78)
Male CEO	95.2%	93.62%	91.89%	92.99%	92.87%	96.7%
	(0.42)	(0.24)	(0.35)	(0.29)	(0.28)	(0.46)
Medical Center	0.19%	0.04%	0.07%	0.18%	0.00%	0.05%
Hospital	5.46%	3.33%	2.6%	3.32%	0.00%	1.00%
FT_opt	1.68	0.95	1.31	1.21	2.20	1.02
	(4.56)	(1.17)	(3.76)	(2.99)	(4.55)	(2.57)
PT_opt	0.67	1.04	1.39	0.93	2.15	0.71
	(1.89)	(2.28)	(6.58)	(2.72)	(5.72)	(1.59)
Obs. No.	1080	2576	4114	38,026	36,765	1261

Note: Standard deviations are in parentheses. Volume is the average number of outpatient consultation services provided in a quarter. FT_opt and PT_opt are the number of full-time and part-time ophthalmologists, respectively, in an institution.

**Table 3:** Regression Analyses of Number of Physician Visits of Patients–Patient-level Data.

	OLS	Poisson	NBReg
Pt. Characteristics			
Male	0.258 ***	1.072 ***	1.072 ***
	(3.03)	(4.28)	(5.75)
Income	0.164 ***	1.044 ***	1.042 ***
	(2.058)	(4.01)	(5.02)
CCI	0.838 ***	1.206 ***	1.211 ***
	(3.028)	(3.005)	(4.13)
Age	−1.512 ***	0.812 ***	0.796 ***
	(4.080)	(−4.012)	(−3.02)
age2	0.089 ***	1.006 ***	1.01 ***
	(3.007)	(2.001)	(3.28)
Ins. Characteristics			
Age_inst	−0.010 *	0.988 ***	0.998 *
	(2.004)	(−4.77)	(−2.13)
Age_inst^2^	0.00 *	1.00 ***	1.00 *
	(1.98)	(3.23)	(1.98)
Med. center	0.472	0.88 **	0.88
	(0.361)	(−1.90)	(−1.36)
Hospital	0.055	0.869 ***	0.98
	(1.247)	(−15.24)	(−0.34)
HHI	−5.697 **	0.99 ***	0.99 **
	(2.298)	(−4.38)	(−2.92)
Parent Employment			
Pub. sector	0.080	0.026	0.923
	(0.16)	(−0.32)	(−0.43)
Private sector	0.060	0.017	0.855
	(0.060)	(−0.011)	(−0.015)
Farmer	−0.289 ***	0.081 ***	0.921 ***
	(4.076)	(−15.014)	(−3.019)
School	0.015	0.002	1.007
	(1.106)	(−0.020)	(0.027)
Self-employed	0.244	0.065	1.055
	(0.221)	(−0.041)	(0.056)
Others	0.007	0.002	1.205
	(1.073)	(−0.014)	(0.018)
Premium	−0.216 ***	0.958 ***	0.995 ***
	(5.040)	(−15.01)	(−5.010)
Reimbursement Categories			
Natural injury	5.134 **	1.011 *	0.902
	(2.146)	(1.916)	(−0.551)
Respirator	18.642 ***	1.317 **	1.264
	(3.242)	(2.427)	(0.691)
Seasonal Effects			
Quarter 2	−0.376 ***	0.102 ***	0.100 ***
	(4.042)	(−2.82)	(−3.11)
Quarter 3	−0.586 ***	0.164 ***	0.161 ***
	(3.42)	(−5.808)	(−5.19)
Quarter 4	−0.106 *	0.028 ***	0.025 *
	(1.843)	(−4.83)	(−2.11)
Point	−0.449	−0.132	−0.116
	(0.43)	(0.082)	(0.11)
Copay	0.001	0.001	0.001
	(0.001)	(0.001)	(0.001)
R^2^	0.12	-	-
Obs.	35,549	35,549	35,549

Note: ***, **, * represent statistical significance at the 1%, 5%, and 10% levels, respectively. Both district and seasonal fixed effects are included in the regressions. The HHI is the Herfindahl-Hirschman index. The CCI is the Charlson comorbidity index. Incidence rate ratios (IRRs) are reported for the Poisson and NB models. OLS Exp is the analysis of myopic care expenditure in natural logarithm as the dependent variable.

**Table 4:** Regression Results for Institution’s Outpatient Visits–Institution-level Data.

	OLS	Poisson	NBReg	OLS_Exp
Inst. Characteristics				
Age	−3.008	0.97 ***	0.99	0.001
	(−0.60)	(−52.16)	(−0.53)	(0.11)
Age^2^	0.125	1.00 ***	1.00	0
	(1.63)	(62.93)	(0.62)	(−0.063)
HHI	2.367	0.90 ***	0.95 ***	−0.02 **
	(0.57)	(−48.99)	(−6.47)	(−2.60)
Med. Center	649.74 ***	2.11 ***	1.51 *	0.95 ***
	(5.99)	(95.51)	(2.14)	(3.44)
Hospital	312.347 *	1.07 ***	0.96	−1.22 ***
	(2.32)	(5.78)	(−0.15)	(−3.71)
CEO_Male	−16.88	0.80 ***	0.85 *	−0.12
	(−0.28)	(−27.12)	(−2.24)	(−1.088)
Inst. Scale				
Outpt Vol.	0.003 ***	1.00 ***	1.00 ***	0.00 ***
	(16.71)	(278.76)	(16.38)	(5.70)
Total no. of	−3.194 ***	−0.99 ***	0.99 ***	−0.004 **
physicians	(−5.798)	(−149.008)	(−11.87)	(−3.277)
FT-Opt.	−98.943	1.95 ***	3.23 ***	0.12
	(−0.446)	46.20	(3.91)	(0.25)
PT-Opt	48.795	1.13 ***	1.52 *	0.002
	(0.37)	(14.27)	(2.45)	(0.01)
Point	−29.53	0.98	1.39	−0.75 ***
	(−0.125)	(−0.677)	(0.68)	(1.12)
Copay	−1.38	0.99 ***	1.00	0.00
	(−1.871)	(−3.394)	(0.58)	(0.001)
Time trend	−17.341 *	0.96 ***	0.93 ***	0.047 **
	(−2.417)	(−52.031)	(−4.99)	(2.42)
Seasonal Effect				
Quarter 2	17.834	0.91 ***	1.07	0.049
	(51.315)	(−10.01)	(−0.79)	(0.055)
Quarter 3	−12.380	0.93 ***	0.91	0.045
	(52.305)	(−9.01)	(−0.58)	(0.057)
Quarter 4	69.811	0.90 ***	1.04	0.110
	(53.562)	(−11.01)	(−1.2)	(0.058)
R^2^	0.67	-	-	0.54
Obs.	733	733	733	733

Note: ***, **, * represent statistical significance at the 1%, 5%, and 10% levels, respectively. FT, PT-opt refer to the numbers of full-time or part-time ophthalmologists in the facility. IRRs (incidence rate ratios) are reported for both Poisson and NBReg models. *Z*-values are in parentheses.

**Table 5:** GLS Regression Analyses for Number of Office Visits–District Aggregate Data.

	Patient Visit (1)	Inst_Service All (2)	Inst_Service Clinic (3)	Inst_Service Hospital (4)
Point Value	−0.278 ***	−22.225 ***	−16.1 ***	−2.20
	(−2.80)	(−5.07)	(−4.35)	(−1.37)
Income	−0.251 ***	−8.631 ***	−7.95 ***	−2.01 **
	(−4.40)	(−3.13)	(−3.53)	(−1.97)
NHCC	−0.005	−4.015 ***	−3.40 ***	−0.67 **
	(−0.36)	(−6.80)	(−6.87)	(−2.41)
Fee	−0.049 ***	−0.408	−0.055	−0.088
	(−2.60)	(−0.48)	(−0.08)	(−0.28)
Copay-1	−0.037	0.189	0.043	0.018
	(−1.54)	(0.18)	(0.05)	(0.05)
Copay-2	−0.112 ***	0.719	0.481	−0.327
	(−3.67)	(0.52)	(0.42)	(−0.64)
Seasonal Effect				
Quarter 2	−0.029 **	−1.525 ***	−1.78 ***	0.189
	(−2.46)	(−2.73)	(−3.21)	−0.78
Quarter 3	−0.038 ***	−1.406 **	−1.72 ***	0.214
	(−3.16)	(−2.47)	(−3.06)	−0.87
Quarter 4	−0.015	−0.571	−0.154	0.244
	(−1.26)	(−1.01)	(−0.28)	−0.99
Density	0.006	4.261 ***	3.380 ***	1.108 ***
	(0.34)	(6.21)	(5.96)	(3.38)
edu_high	−0.008 ***	−0.297 ***	−0.32 ***	0.019
	(−5.55)	(−4.74)	(−5.73)	(0.66)
edu_coll	0.006 ***	0.389 ***	0.36 ***	−0.01
	(3.57)	(5.34)	(5.85)	(−0.33)
Time trend	0.055 ***	0.642 ***	0.690 ***	0.057
	(15.53)	(4.05)	(5.29)	(0.89)
Obs.	1191	1191	1191	1191

Note: ***, ** represent statistical significance at the 1%, 5%, and 10% levels, respectively. Regressions are performed with panel GLS with heteroskedasticity, and AR1 is assumed. NHCC denotes the density of health care facilities, that is, the number of health care facilities per square kilometer in the district. Income represents the district average household income as a natural logarithm. Fee denotes the percentage of income contributed to the NHI as an insurance premium decreed by NHIA. Copays 1 and 2 represent the copayment reform for hospital utilization in September 2002 and the implementation of high surcharges for nonreferral visits in September 2005, respectively. Density denotes the population density of each district, and edu_high and edu_coll denote the percentage of residents who have completed high school or college, respectively.

**Table 6:** GLS Analyses of Outpatient Expenditure (Revenue) by Institution Types–District Aggregate Data.

	Patient Exp (1)	Inst_rev All Level (2)	Inst_rev Clinic (3)	Inst_rev Hospital (4)
Point value	−0.756 ***	−1.438 ***	−0.85 ***	−1.125
	(−2.82)	(−3.83)	(−2.73)	(−0.75)
Income	−0.418 ***	−0.737 ***	−0.64 ***	−0.984
	(−2.75)	(−3.19)	(−3.54)	(−1.20)
NHCC	−0.112 ***	−0.395 ***	−0.35 ***	0.450 **
	(−3.12)	(−8.02)	(−8.92)	(2.02)
Fee	0.002	0.003	0.055	−0.262
	(0.04)	(0.04)	(0.96)	(−0.98)
Copay-1	0.178 ***	0.195 **	0.214 ***	0.015
	(2.80)	(2.19)	(2.93)	(0.04)
Copay-2	0.166 **	0.262 **	0.281 ***	−0.321
	(2.03)	(2.29)	(2.99)	(−0.73)
Seasonal Effect				
Q 2	−0.035	−0.058	−0.074 *	0.115
	(−1.07)	(−1.25)	(−1.64)	−0.55
Q 3	−0.066 **	−0.04	−0.073	0.092
	(−1.98)	(−0.85)	(−1.57)	−0.42
Q 4	−0.036	0.017	0.024	0.243
	(−1.09)	(0.36)	−0.53	−1.15
Density	0.150 ***	0.463 ***	0.394 ***	−0.27
	(3.68)	(7.91)	(8.61)	(−1.06)
edu_high	−0.025 ***	−0.026 ***	−0.03 ***	0.085 ***
	(−5.90)	(−4.69)	(−6.09)	(3.54)
edu_coll	0.009 *	0.027 ***	0.024 ***	−0.06 **
	(1.86)	(4.26)	(4.60)	(−2.46)
Time	0.103 ***	0.048 ***	0.054 ***	0.112 **
trend	(10.80)	(3.61)	(5.01)	(2.29)
Obs.	1191	1191	1191	1191

Note: ***, **, * represent statistical significance at the 1%, 5%, and 10% levels, respectively. Regressions are performed with panel GLS with heteroskedasticity, and AR1 is assumed. NHCC denotes the number of health care facilities in the district. Income is the average district household income as a natural logarithm, and fee denotes the percentage of income contributed to the NHI as an insurance premium. Density denotes the population density of each district, and edu_high and edu_coll denote the percentage of residents who have completed high school or college, respectively. District fixed effects are included in the analyses but are not reported to conserve space.

## Data Availability

The data presented in this study are not publicly available due to privacy concerns and restrictions from the Taiwan MOHW data center.

## Appendix A

**Table A1:** Copayment Changes for Outpatient Services and Medications under Taiwan National Health Insurance.

Time	Medical Center	Regional Hospitals	District Hospitals	Clinics	Drug Fee	High User Surcharge
May 1995	100	100	50	50	None	None
March 1997	150	100	50	50	None	None
August 1999	150	100	50	50	20~100	<49: NT$0; 49~156: NT$50; >156: NT$100;
January 2000	150	100	50	50	20~100	<25: NT$0; 25~156: NT$50; >156: NT$100;
July 2001	150	100	50	50	20~200	<25: NT$0; 25~156: NT$50; >156: NT$100;
September 2002	210	140	50	50	20~200	<25: NT$0; 25~156: NT$50; >156: NT$100;
January 2004	210	140	50	50	20~200	None
July 2005	360	240	80	50	20~200	None

Note: There has been no copayment reform since 2005 through the end of study period. In 1999, high user referred to users who visited physician offices more than 50 times a year. This number decreased to 25 times a year after 2000. Medication copayments are TWD 20 for each TWD 100 of the total drug fee incurred, with a maximum TWD 100 (TWD 200 after 2000) copayment surcharge. All dollar values are in TWD, which trades at approximately USD 1 to TWD 30.
